# Crosstalk between Platelets and the Immune System: Old Systems with New Discoveries

**DOI:** 10.1155/2012/384685

**Published:** 2012-09-12

**Authors:** Conglei Li, June Li, Yan Li, Sean Lang, Issaka Yougbare, Guangheng Zhu, Pingguo Chen, Heyu Ni

**Affiliations:** ^1^Department of Laboratory Medicine and Pathobiology, University of Toronto, Toronto, ON, Canada M5S 1A8; ^2^Department of Laboratory Medicine, Keenan Research Centre, Li Ka Shing Knowledge Institute, St. Michael's Hospital, and Toronto Platelet Immunobiology Group, University of Toronto, Toronto, ON, Canada M5S 1A8; ^3^Canadian Blood Services, Toronto, ON, Canada M5G 2M1; ^4^Department of Medicine and Department of Physiology, University of Toronto, Toronto, ON, Canada M5S 1A8

## Abstract

Platelets are small anucleate cells circulating in the blood. It has been recognized for more than 100 years that platelet adhesion and aggregation at the site of vascular injury are critical events in hemostasis and thrombosis; however, recent studies demonstrated that, in addition to these classic roles, platelets also have important functions in inflammation and the immune response. Platelets contain many proinflammatory molecules and cytokines (e.g., P-selectin, CD40L, IL-1**β**, etc.), which support leukocyte trafficking, modulate immunoglobulin class switch, and germinal center formation. Platelets express several functional Toll-like receptors (TLRs), such as TLR-2, TLR-4, and TLR-9, which may potentially link innate immunity with thrombosis. Interestingly, platelets also contain multiple anti-inflammatory molecules and cytokines (e.g., transforming growth factor-**β** and thrombospondin-1). Emerging evidence also suggests that platelets are involved in lymphatic vessel development by directly interacting with lymphatic endothelial cells through C-type lectin-like receptor 2. Besides the active contributions of platelets to the immune system, platelets are passively targeted in several immune-mediated diseases, such as autoimmune thrombocytopenia, infection-associated thrombocytopenia, and fetal and neonatal alloimmune thrombocytopenia. These data suggest that platelets are important immune cells and may contribute to innate and adaptive immunity under both physiological and pathological conditions.

## 1. Platelets in Hemostasis and Thrombosis: Classical Role and Nonclassical Mechanisms

Platelets, which were first identified around 130 years ago, are small anucleate cells circulating in the blood with a diameter of 1-2 microns [1–5]. They are the second most abundant cells, after red blood cells, in the blood circulation with a normal concentration of 150–400 × 10^9^/L in humans. Platelets are produced from their precursor megakaryocytes in the bone marrow [4–8]; immature larger proplatelets are initially released by megakaryocytes into the blood due to local shear stresses in the bone marrow. These proplatelets may further mature in the lung, although the process is largely unknown [8, 9].

The major physiological role of platelets is to accumulate at sites of damaged blood vessel endothelium and initiate the blood clotting process. Platelet adhesion, activation, and subsequent aggregation at sites of vascular injury are critical to the normal arrest of bleeding [10–12]. When the vessel endothelium is injured, collagen and other subendothelial matrix proteins are exposed allowing platelets in the circulation to bind, which results in platelet activation. Activated platelets release certain intracellular soluble mediators, leading to the recruitment and activation of additional platelets at the injury site [10–12]. This platelet response is one key mechanism required to stop bleeding (i.e., the first wave of hemostasis); the other is the coagulation system, which is initiated by tissue factor (extrinsic) pathway or contact factor (intrinsic) pathway to generate thrombin and polymerized fibrin [10, 13–15]. There are many interactions between these two mechanisms which lead to clotting. For example, platelets (particularly activated platelets) accelerate coagulation by providing a negatively charged phosphatidylserine- (PS-) rich membrane surface that enhances the generation of thrombin, which converts fibrinogen (Fg) to fibrin [16, 17]. Conversely, thrombin generated via the coagulation process is a potent platelet activator [18–20] that induces platelet activation and granule release (e.g., P-selectin translocation to the cell surface). Fibrin (especially polymerized fibrin) also stabilizes the platelet plug [21]. Deficiencies in platelet adhesion/aggregation or coagulation are associated with bleeding disorders [14, 22–25]. However, inappropriate platelet plug formation may also result in thrombosis/vessel obstruction. Unstable angina and myocardial infarction typically result from platelet adhesion/aggregation at ruptured atherosclerotic lesions in coronary arteries. Thrombosis in the coronary or cerebral arteries is the major cause of morbidity and mortality worldwide [26, 27]. In addition, it has been demonstrated that thrombus formation in the placenta can lead to fetal loss during pregnancy in several disease conditions, such as antiphospholipid syndrome and estrogen sulfotransferase deficiency [28–31]. Recently, our group also found in murine models that some maternal antifetal platelet antibodies can cause platelet activation and excessive thrombosis in the placenta, which may lead to miscarriage [17]. Thus, the same processes (platelet adhesion and aggregation) play contrasting but critical roles (physiological, i.e., hemostasis versus pathological, i.e., thrombosis).

### 1.1. Molecular Events of Platelet Adhesion and Aggregation

It is now clear that platelet receptors, GPIIbIIIa (*α*IIb*β*3 integrin) and the GPIb*α* complex, which are two abundant glycoproteins expressed on platelets, play the predominant roles in platelet adhesion and aggregation at the site of vascular injury [10–12]. It has been recognized that platelet GPIb*α*, primarily via binding to immobilized von Willebrand factor (VWF) on collagen, is essential for initiation of platelet-vessel wall interaction, particularly at high shear stress [26, 32, 33]. The GPIb-VWF interaction and/or various platelet agonists (e.g., ADP, thrombin, collagen, and thromboxane A_2_) can cause platelet activation resulting in a conformational change in *α*IIb*β*3 integrin on the platelet surface [34, 35], which allows for ligand binding (e.g., fibrinogen) [35–37]. When bound to activated *α*IIb*β*3 integrin, fibrinogen is able to cross-link adjacent platelets, leading to platelet aggregation and subsequent formation of a platelet plug [38, 39].

Although it has been well documented since the 1960s that Fg is required for platelet aggregation [40], using an FeCl_3_ injury intravital microscopy thrombosis model, we demonstrated that occlusive thrombus formation still occurred in both Fg^−/−^ and Fg/VWF double deficient (Fg/VWF^−/−^) mice [41]. We further demonstrated that Fg/VWF-independent platelet aggregation can be induced in vitro under more physiological conditions (i.e., nonanticoagulated blood) [42]. This concept of Fg- and VWF-independent thrombus formation has been confirmed by several groups in both animal models [43] and human afibrinogenemic patients [44], although the effects of Fg and VWF deficiency on clot formation may be different between mice and humans. In contrast, neither platelet aggregation nor platelet-rich thrombi were observed in *β*3 integrin-deficient (*β*3^−/−^) mice (lacking the *β* subunit of *α*IIb*β*3 and *α*V*β*3 integrins) [41, 45]. These data suggest that nonclassical *β*3 integrin ligands (i.e., not Fg or VWF) exist, which can support robust platelet aggregation independent from Fg and VWF.

### 1.2. Role of Plasma Fibronectin in Thrombosis and Hemostasis

Interestingly, fibronectin, another ligand of *β*3 integrin, is increased 3–5-fold in platelets from either Fg^−/−^ or Fg/VWF^−/−^ mice, although no obvious change in plasma fibronectin levels was observed [41, 46]. We further observed that platelets from an afibrinogenemic patient had enhanced fibronectin content [23]. Our subsequent studies revealed that the increase in fibronectin content in Fg^−/−^ platelets was due to enhanced plasma fibronectin internalization in the absence of Fg, which competitively binds to *β*3 integrin for internalization into platelets [21].

To test whether fibronectin is the ligand-mediating platelet aggregation in Fg/VWF^−/−^ mice, Fg/VWF/pFn triple deficient mice were generated. Surprisingly, we found that platelet aggregation was not abolished but was actually enhanced in Fg/VWF/pFn^−/−^ mice compared to Fg/VWF^−/−^ mice [47], indicating that fibronectin is unlikely the ligand of *β*3 integrin that mediates thrombosis in the absence of both Fg and VWF. Identification and characterization of these novel *β*3 integrin ligands that mediate Fg/VWF-independent platelet aggregation will provide insights into the mechanisms of hemostasis and thrombosis in both normal and gene-deficient human populations and may lead to new targets for antithrombotic therapies.

In addition to their classic roles in hemostasis and thrombosis, recent studies suggest that platelets are also involved in many other physiological and pathophysiological processes, such as inflammation, angiogenesis, and tumor growth [48–51]. Interestingly, although platelets are anucleate cells, they can still de novo synthesize proteins following stimulation by platelet outside-in signalling [52]. We recently demonstrated that interactions between *β*3 integrins and their ligands (e.g., plasma Fg [53] and fibronectin (Andrews M and Ni H, unpublished data)) induced platelet P-selectin synthesis, which may affect not only hemostasis and thrombosis but also inflammation and immune responses. In this paper, we will mainly focus on the interaction between platelets and the immune system.

## 2. The Interaction between Platelets**** and Immune System

In vertebrates, there are two types of immunity to protect the host from infection: innate and adaptive. The innate immune system is genetically programmed to detect invariant features of invading microbial pathogens, while the adaptive immune system employs antigen-specific receptors that are generated de novo in each species [54]. Phagocytosis was first described by Metchnikoff more than a century ago, but research into innate immunity was largely overshadowed by the discovery of antibodies, CD4^+^ and CD8^+^ T cells, and other components of the adaptive immune response [55]. However, the recent discovery of pathogen recognition receptors (PRRs), such as Toll-like receptors (TLRs), Nod-like receptors, and RIG-I-like receptors, which recognize pathogen-associated molecular patterns (PAMPs) that are conserved among microbial pathogens, has greatly advanced our understanding of innate immunity. Platelets express many immunomodulatory molecules (e.g., P-selectin, TLRs, CD40L) and cytokines (e.g., IL-1*β*, TGF-*β*) and have the ability to interact with various immune cells. These properties confer platelets the ability to influence both innate and adaptive immune responses [48]. Alternatively, the immune system (e.g., antibodies, cytokines, immune cells) may target platelets and lead to several immune-mediated diseases, such as autoimmune thrombocytopenia, infection-associated thrombocytopenia, and fetal and neonatal alloimmune thrombocytopenia.

### 2.1. Platelets are Part of the Innate Immune System

Anucleate platelets are found only in mammals. In lower vertebrates, cells involved in hemostasis and blood coagulation are nucleated, termed thrombocytes. In many invertebrates, only one type of cell circulates in the blood, which is responsible for multiple defence mechanisms of the host, including hemostasis and immune functions [56]. Interestingly, mammalian platelets possess many capabilities that are similar to these defensive circulating cells in invertebrates. Platelets contribute to innate immunity in various ways: (1) platelets possess rudimentary antibacterial and phagocytic activity and have been shown to interact with bacteria, viruses, and parasites [57–59]. The interaction of bacteria with platelets induces platelet activation and secretion of antimicrobial peptides [60]; (2) platelets contain many proinflammatory cytokines (e.g., IL-1), which modulate the inflammatory/immune response [48, 49, 61–65]. It has been reported that platelet IL-1*α* drives cerebrovascular inflammation by inducing brain endothelial cell activation and enhancing their release of the chemokine CXCL1 [66]. Platelet-derived IL-1 also stimulates cytokine production (e.g., IL-6 and IL-8) by vascular smooth muscle cells [67]; (3) platelets express several functional Toll-like receptors (TLRs), such as TLR-2, TLR-4, and TLR-9 [68]. By interacting with TLR-4 on platelets, lipopolysaccharide (LPS) from the gram-negative bacteria activates platelets and induces platelet-neutrophil interactions, leading to neutrophil degranulation and release of extracellular traps that can kill the bacteria [69]. It has been demonstrated that LPS-stimulated platelet secretion potentiates platelet aggregation and thrombus formation via a TLR-4/MyD88 pathway, thus linking innate immunity with thrombosis [70]. However, it remains to be determined whether ligand interaction with other platelet TLRs, such as TLR-2 and TLR-9, also enhances thrombus formation; (4) vessel occlusion by thrombotic events in small vessels may play a role in the containment of invasive microorganisms, which prevents spreading of this micropathogen-mediated septicaemia and viraemia and contributes to innate immunity. It also remains unclear whether platelets express other kinds of PRRs, such as Nod-like receptors and RIG-I-like receptors; (5) a recent discovery found that, during malaria infection, platelets can adhere to red blood cells infected with *Plasmodium falciparum* (*P. falciparum*) and induce apoptosis of these intracellular malaria parasites by releasing platelet granule components [71]. Mice deficient in C-mpl (Mpl^−/−^), which have one-tenth as many circulating platelets as wild-type controls [72], are more susceptible to death induced by* P. falciparum* infection. The lethal effects of platelets on *P. falciparum* parasites appear to require platelet activation, since aspirin or other inhibitors of platelet function abrogated these effects [71]. Consistent with these observations, recent studies demonstrated that thrombocytopenia in patients with primary chronic autoimmune thrombocytopenia is associated with a significantly higher long-term risk of infection [73]. Thus, platelets are proinflammatory cells and are an important part of innate immunity.

It is very interesting that platelets also contain multiple anti-inflammatory cytokines (e.g., transforming growth factor-*β*, TGF-*β*). TGF-*β* is a potent immune suppressive factor. It has been reported that metastasizing tumor cells induce platelets to secrete TGF-*β*, which inhibits the antitumor activity of natural killer (NK) cells by downregulating the expression of the activating receptor, natural killer group 2 member D (NKG2D), on NK cells [74]. Neutralization of TGF-*β* in platelet releasate prevented the downregulation of NKG2D on NK cells and restored their antitumor activity [74]. It remains unknown whether malignant tumor cells can drive platelets to release TGF-*β*, which inhibits the function of other tumor-infiltrating lymphocytes (besides NK cells) in the tumor microenvironment, allowing the tumor cells to evade the host's immunosurveillance. It has also been demonstrated that platelets may assist tumor cells in evading immune cells via transfer of major histocompatibility complex (MHC) I from platelets to tumor cells [75]. Furthermore, platelets contain a large amount of thrombospondin-1 (TSP-1), which comprises around 25% of the total protein content of platelet *α*-granules [76]. It has been demonstrated that TSP-1 not only activates the anti-inflammatory cytokine TGF-*β*1 [77] but also inhibits the phagocytic capacity of macrophages. Murine macrophages deficient in TSP-1 have increased phagocytic function and TSP-1-deficient mice are protected from sepsis-associated mortality [78]. TSP-1 expression is significantly elevated on the surface of platelets in sepsis patients [79], and polymorphisms of TSP-1 have been linked with the development of sepsis-related organ failure [78]. Therefore, platelets are likely not only proinflammatory cells but also modulators that balance inflammation and immune responses.

It is intriguing whether a platelet can decide to support or inhibit inflammation since it contains both pro- and anti-inflammatory cytokines. Platelets also contain both angiogenesis inhibitors (e.g., endostatin, angiostatin, TSP-1, and platelet factor 4) and stimulators (e.g., vascular endothelial growth factor, angiopoietin 1, platelet-derived growth factor and insulin-like growth factor). It has been reported that proangiogenic and antiangiogenic molecules are stored in different *α*-granules inside platelets and megakaryocytes [50] and that these molecules are selectively released depending on the pathway of platelet activation [80, 81]. Interestingly, angiogenesis may be seen as an inflammatory response, since the newly formed vessels can transport inflammatory cells to sites of inflamed tissues and supply nutrients and oxygen to enhance proliferation of these tissues [82–85]. Since platelets also contain both proinflammatory (e.g., IL-1*β*) and anti-inflammatory cytokines (TGF-*β* and TSP-1), it remains to be determined whether they are also stored in different platelet granules and whether these cytokines are selectively released depending on local inflammation status.

Platelets also contain many chemokines. It has been demonstrated in vitro that the platelet-derived chemokine platelet factor 4 prevented monocytes from undergoing spontaneous apoptosis and instead induced the differentiation of monocytes into macrophages [86]. However, it remains to be determined whether platelets are involved in modulating the function of activated macrophages, although it is very likely. Considering the abundance of platelets in the circulation, it is reasonable to assume that platelets may act as sentinel cells and sense the invasion of foreign microorganisms via platelet TLRs, and by releasing chemokines, platelets may recruit more inflammatory cells (e.g., neutrophils) to sites of infection. Further studies are necessary to test these possibilities.

### 2.2. Platelets Contribute to Adaptive Immunity

It has been reported that P-selectin (CD62P) plays an important role in the development of the Th-1 immune response [87]. Upon platelet activation, P-selectin is translocated from the *α*-granule to the platelet surface. P-selectin can then interact with peripheral nodes addressin (PNAd) on high endothelial venules (HEV) and P-selectin glycoprotein ligand-1 (PSGL-1) on lymphocytes simultaneously, thus mediating the rolling and recruitment of lymphocytes to HEV of peripheral lymph nodes [88]. Our recent study demonstrated that plasma Fg, which is a key molecule for blood coagulation and platelet aggregation, through interaction with platelet *β*3 integrin, can deliver signals to platelets and induce de novo synthesis of P-selectin, which is required for maintenance of the P-selectin content in platelets [53]. This study provided a new link between coagulation/hemostasis and inflammation/immune responses.

In addition to P-selectin, activated platelets also express CD40L on their surface, which plays an important role in supporting immunoglobulin class switch and augmenting CD8^+^ T-cell function during viral infection [89]. These events can directly affect B-cell differentiation and proliferation, which ultimately affects germinal center formation and antibody production [90]. Thus, platelets are also actively involved in adaptive immunity. Since platelets contain functional TGF-*β*, which is essential for the development of naturally occurring Foxp3^+^ regulatory T cells (nTreg) or T helper 17 cells (Th17) depending on the local cytokine environment [91], it remains unknown whether platelets modulate the balance of immune tolerance and inflammation, contributing to autoimmune diseases.

It has been reported that the effects of platelets on adaptive immunity may play a significant role in the host's defense against bacterial or viral infections. Platelets can actively bind gram-positive bacteria (e.g., *Listeria monocytogenes*) in the circulation and deliver them to splenic CD8*α* ^+^ dendritic cells, promoting antibacterial CD8^+^ T-cell expansion [92]. It has also been reported that platelets contain a large amount of serotonin, which prolongs the activation of CD8^+^ T cells via an unknown mechanism and aggravates virus-induced liver immunopathology [93].

### 2.3. Platelets are Involved in Lymphatic Vascular Development

Blood and vessels have shared developmental origins and function together to nourish the developing embryo [94, 95]. Definitive hematopoietic stem cells are derived from a subset of endothelial cells of the dorsal aorta in the aorta-gonad-mesonephros region of the developing embryo [96, 97]. The lymphatic vessels are a specialized vascular system that is parallel but separate from the blood vessels [98]. They form an extensive collecting network that maintains tissue fluid homeostasis, absorbs dietary lipids in the intestine, and facilitates immune cell trafficking and surveillance [99]. These specialized vessels are mainly composed of lymphatic endothelial cells (LECs) that originate in the cardinal vein [98]. During embryonic development, LECs migrate away from the cardinal vein and assemble into vessels to form a de novo collecting vascular system [98, 100]. It has been demonstrated that this blood-lymphatic vessel separation is regulated by a SYK-SLP-76 signalling pathway in blood cells, but the cell type and molecular regulation mechanisms were not well understood [99, 101]. Recently, several independent groups have identified platelets as the cell type in which SLP-76 signalling is essential to regulate lymphatic vessel development [101–103]. It was found that platelet C-type lectin-like receptor 2 (CLEC-2) can bind podoplanin (PDPN) on the surface of lymphatic endothelial cells and activate SLP-76 signalling to mediate blood and lymphatic vessel separation during embryonic development [101]. This interaction is required for blood-lymphatic separation in nonhematopoietic cells. This mechanism is further confirmed by the findings that platelet-specific deletion of SLP-76 is sufficient to induce a phenotype in which blood and lymphatic vessels commingle and that platelet-deficient embryos also exhibit the same phenotype [101, 102]. Considering the versatility of platelets, it would not be surprising if platelets play additional roles in embryonic/fetal development [104], including possible contributions to the development of the immune system.

## 3. Pathogenesis of Immune-Mediated**** Thrombocytopenia

Thrombocytopenia is a disorder in which the number of circulating platelets is abnormally low (<150 × 10^9^/L). Decreased platelet counts may lead to a severe bleeding diathesis, which, in some cases, may be life-threatening [48]. There are several major categories of thrombocytopenia, grouped according to the cause of the disease: (1) immune-mediated thrombocytopenia, (2) genetic deficiency-associated thrombocytopenia, and (3) malignancy-associated thrombocytopenia, which may occur in diseases such as chronic lymphocytic leukemia and lymphomas, prostate, breast, and ovarian cancers [105–109].

Immune-mediated thrombocytopenia can be further divided into autoimmune and alloimmune thrombocytopenia. Autoimmune thrombocytopenia is due to an abnormal immune response which develops against one's own platelets. The main types of autoimmune thrombocytopenia are primary immune thrombocytopenia (ITP; termed by a group of researchers in ITP) [110], infection-associated thrombocytopenia and drug-induced thrombocytopenia [111]. Alloimmune thrombocytopenia is due to alloantibody-mediated platelet destruction, in which antiplatelet alloantibodies develop following an immune response against transfused platelets from genetically different donors (termed posttransfusion purpura; PTP) or against paternally-derived alloantigens on fetal platelets during pregnancy (termed fetal and neonatal alloimmune thrombocytopenia (FNAIT or FNIT)) [112].

### 3.1. Platelets in Autoimmune Thrombocytopenia

#### 3.1.1. Autoimmune Thrombocytopenia/Immune Thrombocytopenia (ITP)

Autoimmune thrombocytopenia (ITP) is a bleeding disorder characterized by autoantibody-mediated platelet destruction and impaired platelet production, with an increased bleeding diathesis [113, 114]. The incidence of ITP has been estimated at around 1–2.4 per 10,000 persons [115]. The characteristic of primary ITP is isolated thrombocytopenia (platelet count < 100 × 10^9^/L), in the absence of other conditions that may be associated with thrombocytopenia [110].

To date, the exact mechanisms of primary ITP are unclear [116]. Proposed mechanisms include both enhanced platelet destruction and impaired platelet production [117–122]. The GPIIbIIIa and GPIb*α* complex are the two major antigens targeted by autoantibodies in ITP. In adult ITP patients, approximately 70% of platelet autoantibodies are directed against GPIIbIIIa, and about 20–40% have specificity for the GPIb*α* complex, or both [123]. The antiplatelet antibodies in ITP may accelerate platelet clearance by Fc*γ*-receptor (Fc*γ*R)-bearing macrophages of the reticuloendothelial system, particularly those in the spleen. The autoantibodies in ITP patients may also affect platelet function, via effects on the binding of ligands (e.g., Fg and VWF) to platelet surface receptors [124, 125], which may further enhance the severity of bleeding in these patients. Furthermore, recent studies demonstrated that antiplatelet antibodies from ITP patients suppress megakaryocyte development and induce megakaryocyte apoptosis, thus inhibiting platelet production [121, 122, 126, 127]. Interestingly, there are some ITP patients who are thrombocytopenic but do not have detectable antiplatelet autoantibodies. It is currently unknown whether some low-affinity antibodies exist in these patients or their antibodies recognize conformation-dependent epitopes that are lost during current monoclonal platelet antigen capture assays (MAIPA) procedures. It has also been suggested that in this antibody-negative group of ITP patients, cytotoxic CD8^+^ T cells might be involved in the pathogenesis [128, 129].

To study the pathophysiology of primary ITP, as well as the efficacy and mechanisms of therapies, several laboratory-induced animal models of ITP have been established, such as (NZW × BXSB) F1 mice [130], the antiplatelet antibody passive-transfer model [131, 132], and the splenocyte-engraftment active ITP model [133]. The passive model of ITP was established by the injection of antiplatelet serum or monoclonal antibodies into recipient mice, in which short-term thrombocytopenia subsequently developed [134]. Using this model, it was demonstrated by Nieswandt and his colleagues that rat anti-mouse anti-GPIb*α* monoclonal antibodies (mAbs), but not anti-GPIIbIIIa mAbs, may cause thrombocytopenia in an Fc-independent manner [132]. We further demonstrated that these anti-GPIb*α* mAbs were able to cause thrombocytopenia in Fc receptor *γ*-chain-deficient mice, suggesting that anti-GPIb*α*-mediated thrombocytopenia may occur via an Fc-independent pathway [135]. Intravenous IgG (IVIG), prepared from pooled plasma from more than 1,000 healthy donors, has been considered a first-line therapy for ITP patients [136]. We found that IVIG effectively ameliorated thrombocytopenia induced by all anti-GPIIbIIIa mAbs, but not anti-GPIb*α* mAbs (with the exception of the anti-GPIb*α* antibody p0p4) [135]. Retrospective studies in ITP patients support these data. Go et al. demonstrated that IVIG was an effective therapy in ITP patients without anti-GPIb*α* antibodies (7/7), whereas most patients with anti-GPIb*α* antibodies were refractory to this treatment (7/10) [137]. More recently, Peng et al. observed that approximately 82% of ITP patients with anti-GPIIbIIIa antibodies responded to IVIG therapy, while only 49% of the patients with anti-GPIb*α* antibodies were responsive [138]. Taken together, these data suggest that IVIG is less effective in attenuating ITP mediated by anti-GPIb*α* antibodies compared to anti-GPIIbIIIa antibodies. Steroid treatment is the most commonly used first-line therapy for ITP patients worldwide. Our recent retrospective study demonstrated that ITP patients with anti-GPIIbIIIa antibodies had a 2-3-fold greater response to steroid treatment than patients with anti-GPIb*α* antibodies [139]. This is the first study in which a difference in therapeutic responsiveness to steroids was revealed between anti-GPIIbIIIa- and anti-GPIb*α*-mediated ITP, and the mechanisms underlying this difference are currently under investigation.

The major shortcomings of the passive antiplatelet antibody transfer model of ITP are that this model does not mimic primary chronic (i.e., long-term) ITP, and therefore, it is not feasible to investigate how antiplatelet autoimmunity initiates. To better accommodate these requirements, we have recently established a novel active animal model of ITP [133]. In this model, *β*3^−/−^ mice were transfused with wild-type (WT) platelets, and spleen cells from these immunized *β*3^−/−^ mice were engrafted into irradiated severe combined immunodeficiency (SCID) mice. Platelet counts and bleeding phenotypes were monitored in the SCID recipients. By depleting specific groups of lymphocytes prior to the transfer of splenocytes, CD19^+^ B-cell- (antibody-) and CD8^+^ T-cell- (cytotoxic T-cell-) mediated platelet destruction was observed. Both of these types of immune-mediated platelet destruction were dependent on the presence of CD4^+^ T cells, as CD4^+^ T-cell depletion completely abrogated the ability of the *β*3 integrin-reactive splenocytes to induce severe thrombocytopenia or bleeding symptoms, suggesting that CD4^+^ helper T cells may be responsible for the initiation of immunopathology in ITP [133]. Differences in the responsiveness to IVIG treatment were observed between antibody- and cell-mediated thrombocytopenia, as antibody-mediated thrombocytopenia and associated bleeding were ameliorated by IVIG, while cytotoxic T-cell-mediated severe ITP failed to respond to this therapy [133]. However, the clinical relevance of this study remains to be determined.

#### 3.1.2. Platelets in Infection-Associated Thrombocytopenia

Chronic infections, such as *Helicobacter pylori* (*H. pylori*) [140], human immunodeficiency virus (HIV) [111, 141, 142], hepatitis virus [143, 144], Epstein-Barr virus [145], cytomegalovirus [146], rubella virus [147], and the recently discovered novel Bunyavirus [148], have been linked with secondary ITP. Thrombocytopenia in these infections has been mainly attributed to antigenic mimicry, whereby antibodies targeting the micropathogens cross-react with platelet glycoproteins resulting in accelerated platelet clearance [111, 149].

The gram-negative bacterium *H. pylori*, which colonizes the stomach, has been associated with many gastrointestinal disorders, such as gastritis and gastric adenocarcinoma [150, 151]. Recent studies demonstrated that *H. pylori* infection is also implicated in the pathogenesis of secondary ITP [140], and that eradication of *H. pylori* infection may lead to disease regression [152, 153]. The prevalence of *H. pylori* infection among ITP patients varies by geographical area, ranging from 22% in North America to 85% in Japan [111, 154, 155]. The pathogenesis of *H. pylori*-associated ITP is not well understood, although antigenic mimicry has been implicated [111].

Human immunodeficiency virus (HIV) was first discovered as the cause of acquired immune deficiency syndrome (AIDS). Thrombocytopenia has been observed in HIV-infected patients with an estimated incidence of 4% to 24% [111, 141, 142] and the rate of thrombocytopenia has been strongly associated with the stage of AIDS progression[156]. Most antiplatelet antibodies isolated from HIV-infected patients react with the *β*3 integrin (GPIIIa)-(49–66) peptide, suggesting that GPIIIa-(49–66) is a major antigenic determinant for antiplatelet antibodies in these patients [157]. These anti-GPIIIa-(49–66) antibodies, which cross-react with many HIV proteins (e.g., nef, gag, env, and pol) [149], cause platelet destruction via the induction of reactive oxygen species, independent of complement activation [158].

Hepatitis C virus (HCV) infection has also been linked with thrombocytopenia, and the prevalence of HCV in ITP patients varies from 10% to 36% [143, 144]. Mechanistically, HCV core protein 1 has been shown to induce the generation of antibodies that cross-react with GPIIIa-(49–66), leading to platelet destruction [159]. However, it has been reported that sequestration of platelets in the enlarged spleen (due to portal hypertension and decreased production of thrombopoietin) may also contribute to the pathogenesis of HCV-associated thrombocytopenia [160, 161].

### 3.2. Platelets in Alloimmune Thrombocytopenia

#### 3.2.1. Fetal and Neonatal Alloimmune Thrombocytopenia

Fetal and neonatal alloimmune thrombocytopenia (FNAIT or FNIT) is a life-threatening alloimmune disorder, which results from fetal platelet opsonization and destruction by maternal antibodies that develop during pregnancy [162–165]. The maternal immune system targets paternally derived antigens on fetal platelets, due to platelet gene polymorphisms and generates alloantibodies. These maternal alloantibodies can cross the placenta and enter the fetal circulation. The neonatal Fc receptor (FcRn) has been implicated in mediating the maternofetal transfer of IgG [166, 167]. The maternal alloantibodies bind to fetal or neonatal platelets and cause their destruction. The mechanisms of fetal or neonatal platelet destruction in FNAIT are not well understood, although it is thought that these mechanisms may be similar to those in ITP [168].

FNAIT is the most common cause of severe thrombocytopenia in liveborn neonates. The incidence of FNAIT has been estimated at 0.5–1.5 per 1,000 liveborn neonates [169–172]; however, this number does not include miscarriage caused by the disease, since the rate of fetal mortality in affected pregnant women has not been adequately studied, although miscarriage has been reported by several groups [173–177]. Furthermore, the mechanisms of miscarriage and the potential therapies to prevent miscarriage in these women are unknown. The major risk of FNAIT is severe bleeding, especially intracranial hemorrhage (ICH), which may lead to neurological impairment or death in affected fetuses and neonates [162–165]. Contrary to haemolytic disease of the newborn, almost half of FNAIT cases occur during the first pregnancy [174], thus challenging current diagnostic and therapeutic methods. Furthermore, the rate of recurrence among subsequent platelet antigen-positive siblings is close to 100%, with subsequently affected siblings having either a similar or more severe degree of thrombocytopenia [165, 178].

Similar to ITP, the major antigen target in patients with FNAIT is GPIIbIIIa, as most reported FNAIT cases (around 75%) have been characterized by maternal alloantibodies to human platelet antigen-1a (HPA-1a), which is located on fetal *β*3 integrin [179, 180]. However, there are very few reported cases of FNAIT associated with anti-GPIb*α* antibodies [181–186], which is in clear contrast to the 20–40% prevalence of anti-GPIb*α* complex antibodies in patients with ITP [187–189]. The underlying reason for the surprisingly low incidence of FNAIT mediated by anti-GPIb*α* antibodies has not been explored and the maternal immune responses to fetal platelet antigens remain to be elucidated.

Recent studies suggest that *β*3 integrin is expressed on human placental syncytiotrophoblast cells as early as the first trimester of pregnancy [190, 191]. Thus, the maternal immune system may mount an immune response against “platelet” antigens on placental syncytiotrophoblasts or on fetal platelets that “leak” into the maternal circulation through fetomaternal hemorrhage during pregnancy [112, 190, 191]. Approximately 2–2.5% of the Caucasian population is HPA-1a negative, and maternal alloantibodies to HPA-1a can be formed in homozygous HPA-1bb women carrying a HPA-1a-positive fetus. However, it has been reported that only around 10% of HPA-1a negative pregnant women carrying an HPA-1a-positive fetus will generate anti-HPA-1a antibodies [179]. The human leukocyte antigen- (HLA-) DRB3*0101 allele, which encodes MHC molecule DR52a, has been associated with anti-HPA-1a antibody generation, suggesting that it may be important in presenting the HPA-1a antigen to CD4^+^ T cells that can provide cytokines to “help” B cells to generate anti-HPA-1a antibodies [179, 192]. It has been further demonstrated that HPA-1a, but not HPA-1b, binds to DR52a and forms stable complexes, which can potentially stimulate the response of CD4^+^ T cells [193–195]. By culturing peripheral blood mononuclear cells in the presence of the HPA-1a peptide, HPA-1a-specific CD4^+^ T cells have been isolated from HPA-1a-immunized women that give birth to children with FNAIT, indicating that the T-cell response is involved in the pathogenesis of FNAIT [195, 196].

Our laboratory has recently established animal models of FNAIT using *β*3^−/−^ mice. *β*3^−/−^ female mice were transfused with wild-type (WT) platelets before breeding with WT males [197]. The fetuses from this breeding are *β*3 heterozygous (i.e., antigen positive) and are expected to be targeted by the maternal immune response during pregnancy. We first demonstrated that *β*3^−/−^ mice generated specific antibodies against platelet *β*3 integrin after WT platelet transfusions [197]. The immunized *β*3^−/−^ females were bred with WT males, and we found that neonatal thrombocytopenia and severe bleeding symptoms (e.g., ICH) were observed in the heterozygous pups from immunized *β*3^−/−^ mothers, which recapitulated FNAIT in humans [197]. We found that maternal administration of IVIG during pregnancy downregulated the pathogenic anti-*β*3 antibody levels in both the maternal and fetal/neonatal circulation and markedly ameliorated FNAIT symptoms [197]. Our laboratory has demonstrated that anti-*β*3 antibodies generated in our anti-*β*3-mediated FNAIT model may cross-react with *β*3 integrin on fetal endothelial cells and inhibit proliferation and vascular-like tube formation by endothelial cells in vitro [198].

To investigate the potential reasons for the rarity of reported cases of anti-GPIb*α*-mediated FNAIT in humans, we developed another animal model of FNAIT using GPIb*α* ^−/−^ mice and compared the pathogenesis with our anti-*β*3 integrin-mediated FNAIT model [17, 199]. We found, unexpectedly, that miscarriage occurred in most of the anti-GPIb*α*-mediated FNAIT, which is far more frequent than that mediated by anti-*β*3 antibodies. Mothers with anti-GPIb*α* antibodies exhibited extensive fibrin deposition and apoptosis/necrosis in their placentas, which severely impaired placental function. We further demonstrated, for the first time, that anti-GPIb*α* (but not anti-*β*3) antisera activated platelets and enhanced fibrin formation in vitro and thrombus formation in vivo [17]. Furthermore, anti-GPIb*α* antibodies purified from the anti-GPIb*α* antisera inhibited the binding of *α*-thrombin to platelet GPIb*α*, which may lead to increased free circulating thrombin. This thrombin can convert fibrinogen into fibrin and activate platelets via protease-activated receptors 1 and 4, which may further enhance thrombus formation. Thus, the maternal immune response to fetal GPIb*α* may cause a previously unidentified, nonclassical FNAIT (i.e., spontaneous miscarriage but not neonatal bleeding), which may mask the severity and frequency of anti-GPIb*α*-mediated FNAIT in humans [17, 199]. We demonstrated that IVIG or anti-FcRn therapy efficiently prevented this life-threatening disease, suggesting potential therapeutic interventions for human FNAIT patients affected by miscarriage [17]. The efficacy of IVIG in anti-GPIb*α*-mediated FNAIT may be due to IVIG-mediated downregulation of maternal pathogenic antibodies and blockade of these maternal antibodies from crossing the placenta during pregnancy (via occupancy of FcRn). It is currently unknown whether the maternal immune response against fetal GPIb*α* is indeed a significant cause of miscarriage in humans. Screening the polymorphisms of GPIb*α* (e.g., HPA-2) in women suffering from miscarriage and habitual abortion and detecting anti-GPIb*α* antibodies during pregnancy may provide important information to address this critical question. If anti-GPIb*α* antibodies are indeed a risk factor for miscarriage in humans, identifying these women and treating them during early in pregnancy with IVIG or anti-FcRn therapies may be able to prevent this nonclassical, but devastating, FNAIT.

To further characterize the role of FcRn in the pathogenesis of FNAIT, we developed another murine model of FNAIT using *β*3/FcRn double deficient (*β*3^−/−^/FcRn^−/−^) mice [200, 201]. In this model, we transfused *β*3^−/−^/FcRn^−/−^ female mice with *β*3^+/+^/FcRn^−/−^ platelets and bred them with *β*3^+/+^/FcRn^−/−^ male mice [200]. We observed that the transfused *β*3^−/−^/FcRn^−/−^ mice generated specific antibodies against platelet *β*3 integrin and maintained these antibodies at levels that sufficiently induced thrombocytopenia in adult *β*3^+/+^/FcRn^−/−^ mice. However, no FNAIT developed in these *β*3^−/−^/FcRn^−/−^ female mice when bred with *β*3^+/+^/FcRn^−/−^ males, suggesting that FcRn is essential for the induction of FNAIT [200]. Using our newly generated mouse anti-mouse *β*3 integrin antibodies, we further demonstrated that FcRn is required for the transfer of all IgG isotypes from the maternal circulation to the fetus. Although fetal and maternal sides of the placenta both express FcRn, we found that fetal, but not maternal, FcRn was required for transplacental transfer of anti-*β*3 integrin IgG and the induction of FNAIT. This finding may have broad implications for the basic understanding of how maternal antibodies are transported across the placenta and for infectious diseases [200]. We found that anti-FcRn is a more efficient therapy than IVIG, since the dose of anti-FcRn required for therapeutic efficacy in this FNAIT model is much lower than that of IVIG (at least 200-fold less) [200]. In addition, anti-FcRn has the advantage of not being prepared from human plasma (as is IVIG), thus having less chances of transmitting blood-borne micropathogens. Furthermore, our data suggest that anti-FcRn may be useful to prevent the transplacental transport of maternal pathogenic antibodies in other alloimmune diseases, including alloimmune neonatal neutropenia and haemolytic disease of the newborn, or pathogenic antibody transfer from mothers with autoimmune diseases, such as ITP, systemic lupus erythematosus, and autoimmune haemolytic anemia [200].

#### 3.2.2. Posttransfusion Purpura

Posttransfusion purpura (PTP) is a rare but severe alloimmune complication that usually occurs within 7–10 days following a blood transfusion [112, 202]. The true incidence of PTP is unclear, since this disorder is frequently underdiagnosed or misdiagnosed [112]. Patients with PTP are initially sensitized against the offending platelet antigens either during pregnancy or a blood product transfusion. Upon reexposure to the same platelet antigens (typically via a blood transfusion), the antiplatelet alloimmune response is further stimulated and a sudden rise in antiplatelet antibody titers occurs leading to rapid clearance of both transfused platelets and the patient's endogenous platelets. The mechanisms leading to the destruction of the patient's own antigen-negative platelets are unclear [112]. On most occasions, PTP patients present with sudden onset of widespread purpura following a blood transfusion, but life-threatening bleeding symptoms, such as ICH, have been described in some severe cases, sometimes with a fatal outcome [112]. Although it has been reported that some PTP patients recover spontaneously, therapeutic interventions may be required for patients with severe thrombocytopenia and resulting bleeding diathesis. The administration of IVIG, with or without corticosteroids, has been considered as the first-line therapy for PTP [112, 202].

## 4. Concluding Remarks

It is well known that platelets play critical roles in hemostasis and thrombosis. However, exciting recent studies have revealed many new roles of platelets, such as inflammation/immune responses, tumour growth and metastasis, angiogenesis, and so forth. These new data have revolutionized our understanding of platelet functions. Platelets contain many immunologically functional molecules and contribute to both innate and adaptive immunity, which establishes platelets as immune cells. Considering their abundance in the blood, platelets may act as sentinels to identify invading microorganisms through platelet TLRs. In addition, platelets are also involved in lymphatic vessel development. In addition to the active contributions of platelets to immunity, platelets are also passively targeted in several immune-mediated diseases, although the pathogenesis is not well understood. It remains unknown whether platelets themselves contribute to the initiation or development of these platelet-targeted immune-mediated diseases. Further exploration of the interaction between platelets and the immune system may provide insights into autoimmune diseases, including the chronic process of atherosclerosis, and lead to the development of new therapies to control diseases such as infection and malignant tumors, as well as bleeding disorders.

## References

[1] Coller B (2007). *A Brief History of Ideas about Platelets in Health and Disease, Platelets*.

[2] Oler W, Schaefer E (1873). Ueber einige im Blute vorhandene bakterienbildende Massen. *Centralblatt Für Die Medicinischen Wissenschaften*.

[3] Oler W (1874). An account of certain organisms occurring in the liquor sanguinis. *Proceedings of the Royal Society of London*.

[4] Bizzozero G (1881). Su di un nuovo elemento morfologico del sangue dei mammiferi e della sua importanza nella trombosi e nella coagulazione. *L'Osservatore*.

[5] Bizzozero J (1882). Ueber einen neuen formbestandtheil des blutes und dessen rolle bei der thrombose und der blutgerinnung—untersuchungen. *Archiv für Pathologische Anatomie und Physiologie und für Klinische Medicin*.

[6] Wright J (1906). The origin and nature of blood plates. *The Boston Medical and Surgical Journal*.

[7] Italiano JE, Lecine P, Shivdasani RA, Hartwig JH (1999). Blood platelets are assembled principally at the ends of proplatelet processes produced by differentiated megakaryocytes. *Journal of Cell Biology*.

[8] Junt T, Schulze H, Chen Z (2007). Dynamic visualization of thrombopoiesis within bone marrow. *Science*.

[9] Fuentes R, Wang Y, Hirsch J (2010). Infusion of mature megakaryocytes into mice yields functional platelets. *Journal of Clinical Investigation*.

[10] Ruggeri ZM (1997). Mechanisms initiating platelet thrombus formation. *Thrombosis and Haemostasis*.

[11] Phillips DR, Charo IF, Scarborough RM (1991). GPIIb-IIIa: the responsive integrin. *Cell*.

[12] Andrews RK, Berndt MC (2004). Platelet physiology and thrombosis. *Thrombosis Research*.

[13] Mann KG (2003). Thrombin formation. *Chest*.

[14] Ni H, Freedman J (2003). Platelets in hemostasis and thrombosis: role of integrins and their ligands. *Transfusion and Apheresis Science*.

[15] Hatton MWC, Ross B, Southward SMR, Timleck-DeReske M, Richardson M (2002). Platelet and fibrinogen turnover at the exposed subendothelium measured over 1 year after a balloon catheter de-endothelializing injury to the rabbit aorta: thrombotic eruption at the late re-endothelialization stage. *Atherosclerosis*.

[16] Shen GX (2006). Inhibition of thrombin: relevance to anti-thrombosis strategy. *Frontiers in Bioscience*.

[17] Li C, Piran S, Chen P (2011). The maternal immune response to fetal platelet GPIbalpha causes frequent miscarriage in mice that can be prevented by intravenous IgG and anti-FcRn therapies. *The Journal of Clinical Investigation*.

[18] Sambrano GR, Weiss EJ, Zheng YW, Huang W, Coughlin SR (2001). Role of thrombin signalling in platelets in haemostasis and thrombosis. *Nature*.

[19] Kahn ML, Zheng YW, Huang W (1998). A dual thrombin receptor system for platelet activation. *Nature*.

[20] Vu TKH, Hung DT, Wheaton VI, Coughlin SR (1991). Molecular cloning of a functional thrombin receptor reveals a novel proteolytic mechanism of receptor activation. *Cell*.

[21] Ni H, Papalia JM, Degen JL, Wagner DD (2003). Control of thrombus embolization and fibronectin internalization by integrin *α*IIb*β*3 engagement of the fibrinogen *γ* chain. *Blood*.

[22] Lillicrap D (2009). Genotype/phenotype association in von Willebrand disease: is the glass half full or empty?. *Journal of Thrombosis and Haemostasis*.

[23] Zhai Z, Wu J, Xu X (2007). Fibrinogen controls human platelet fibronectin internalization and cell-surface retention. *Journal of Thrombosis and Haemostasis*.

[24] Gui T, Reheman A, Funkhouser WK (2007). In vivo response to vascular injury in the absence of factor IX: examination in factor IX knockout mice. *Thrombosis Research*.

[25] Gui T, Reheman A, Ni H (2009). Abnormal hemostasis in a knock-in mouse carrying a variant of factor IX with impaired binding to collagen type IV. *Journal of Thrombosis and Haemostasis*.

[26] Ruggeri ZM (2002). Platelets in atherothrombosis. *Nature Medicine*.

[27] Falati S, Gross P, Merrill-skoloff G, Furie BC, Furie B (2002). Real time in vivo imaging of platelets, tissue factor and fibrin during arterial thrombus formation in the mouse. *Nature Medicine*.

[28] Lynch A, Marlar R, Murphy J (1994). Antiphospholipid antibodies in predicting adverse pregnancy outcome: a prospective study. *Annals of Internal Medicine*.

[29] Hughes GRV (1983). Thrombosis, abortion, cerebral disease, and the lupus anticoagulant. *British Medical Journal*.

[30] Bates SM (2010). Consultative hematology: the pregnant patient pregnancy loss. *Hematology*.

[31] Tong MH, Jiang H, Liu P, Lawson JA, Brass LF, Song WC (2005). Spontaneous fetal loss caused by placental thrombosis in estrogen sulfotransferase-deficient mice. *Nature Medicine*.

[32] Ruggeri ZM (2003). Von Willebrand factor, platelets and endothelial cell interactions. *Journal of Thrombosis and Haemostasis*.

[33] Bergmeier W, Piffath CL, Goerge T (2006). The role of platelet adhesion receptor GPIb*α* far exceeds that of its main ligand, von Willebrand factor, in arterial thrombosis. *Proceedings of the National Academy of Sciences of the United States of America*.

[34] Moroi M, Jung SM, Shinmyozu K, Tomiyama Y, Ordinas A, Diaz-Ricart M (1996). Analysis of platelet adhesion to a collagen-coated surface under flow conditions: the involvement of glycoprotein VI in the platelet adhesion. *Blood*.

[35] Xia T, Takagi J, Coller BS, Wang JH, Springer TA (2004). Structural basis for allostery in integrins and binding to fibrinogen-mimetic therapeutics. *Nature*.

[36] Shattil SJ, Hoxie JA, Cunningham M, Brass LF (1985). Changes in the platelet membrane glycoprotein IIb.IIIa complex during platelet activation. *Journal of Biological Chemistry*.

[37] Xiong JP, Stehle T, Diefenbach B (2001). Crystal structure of the extracettutar segment of integrin *α*V*β*3. *Science*.

[38] Ruggeri ZM (2000). Old concepts and new developments in the study of platelet aggregation. *Journal of Clinical Investigation*.

[39] Frojmovic MM (1998). Platelet aggregation in flow: differential roles for adhesive receptors and ligands. *American Heart Journal*.

[40] Cross MJ (1964). Effect of fibrinogen on the aggregation of platelets by adenosine diphosphate. *Thrombosis et Diathesis Haemorrhagica*.

[41] Ni H, Denis CV, Subbarao S (2000). Persistence of platelet thrombus formation in arterioles of mice lacking both von Willebrand factor and fibrinogen. *Journal of Clinical Investigation*.

[42] Yang H, Reheman A, Chen P (2006). Fibrinogen and von willebrand factor-independent platelet aggregation in vitro and in vivo. *Journal of Thrombosis and Haemostasis*.

[43] Jiroušková M, Chereshnev I, Väänänen H, Degen JL, Coller BS (2004). Antibody blockade or mutation of the fibrinogen *γ*-chain C-terminus is more effective in inhibiting murine arterial thrombus formation than complete absence of fibrinogen. *Blood*.

[44] Tsuji S, Sugimoto M, Miyata S, Kuwahara M, Kinoshita S, Yoshioka A (1999). Real-time analysis of mural thrombus formation in various platelet aggregation disorders: distinct shear-dependent roles of platelet receptors and adhesive proteins under flow. *Blood*.

[45] Hynes RO, Hodivala-Dilke KM (1999). Insights and questions arising from studies of a mouse model of Glanzmann thrombasthenia. *Thrombosis and Haemostasis*.

[46] Ni H (2006). Unveiling the new face of fibronectin in thrombosis and hemostasis. *Journal of Thrombosis and Haemostasis*.

[47] Reheman A, Yang H, Zhu G (2009). Plasma fibronectin depletion enhances platelet aggregation and thrombus formation in mice lacking fibrinogen and von Willebrand factor. *Blood*.

[48] Semple JW, Italiano JE, Freedman J (2011). Platelets and the immune continuum. *Nature Reviews Immunology*.

[49] Von Hundelshausen P, Weber C (2007). Platelets as immune cells: bridging inflammation and cardiovascular disease. *Circulation Research*.

[50] Italiano JE, Richardson JL, Patel-Hett S (2008). Angiogenesis is regulated by a novel mechanism: pro- and antiangiogenic proteins are organized into separate platelet *α* granules and differentially released. *Blood*.

[51] Ho-Tin-Noé B, Goerge T, Cifuni SM, Duerschmied D, Wagner DD (2008). Platelet granule secretion continuously prevents intratumor hemorrhage. *Cancer Research*.

[52] Zimmerman GA, Weyrich AS (2008). Signal-dependent protein synthesis by activated platelets: new pathways to altered phenotype and function. *Arteriosclerosis, Thrombosis, and Vascular Biology*.

[53] Yang H, Lang S, Zhai Z (2009). Fibrinogen is required for maintenance of platelet intracellular and cell-surface P-selectin expression. *Blood*.

[54] Iwasaki A, Medzhitov R (2010). Regulation of adaptive immunity by the innate immune system. *Science*.

[55] Silverstein AM (2003). Darwinism and immunology: from Metchnikoff to Burnet. *Nature Immunology*.

[56] Hose J, Martin G, Gerard A (1990). A decapod hemocyte classification scheme integrating morphology, cytochemistry, and function. *The Biological Bulletin*.

[57] Youssefian T, Drouin A, Massé JM, Guichard J, Cramer EM (2002). Host defense role of platelets: engulfment of HIV and *Staphylococcus aureus* occurs in a specific subcellular compartment and is enhanced by platelet activation. *Blood*.

[58] Joseph M, Auriault C, Capron A, Vorng H, Viens P (1983). A new function for platelets: IgE-dependent killing of schistosomes. *Nature*.

[59] Kraemer BF, Campbell RA, Schwertz H (2011). Novel anti-bacterial activities of beta-defensin 1 in human platelets: suppression of pathogen growth and signaling of neutrophil extracellular trap formation. *PLoS Pathogens*.

[60] Cox D, Kerrigan SW, Watson SP (2011). Platelets and the innate immune system: mechanisms of bacterial-induced platelet activation. *Journal of Thrombosis and Haemostasis*.

[61] Wagner DD, Burger PC (2003). Platelets in inflammation and thrombosis. *Arteriosclerosis, Thrombosis, and Vascular Biology*.

[62] von Hundelshausen P, Koenen RR, Weber C (2009). Platelet-mediated enhancement of leukocyte adhesion. *Microcirculation*.

[63] Henn V, Slupsky JR, Gräfe M (1998). CD40 ligand on activated platelets triggers an inflammatory reaction of endothelial cells. *Nature*.

[64] Semple JW, Freedman J (2010). Platelets and innate immunity. *Cellular and Molecular Life Sciences*.

[65] Hawrylowicz CM, Santoro SA, Platt FM, Unanue ER (1989). Activated platelets express IL-1 activity. *Journal of Immunology*.

[66] Thornton P, McColl BW, Greenhalgh A, Denes A, Allan SM, Rothwell NJ (2010). Platelet interleukin-1*α* drives cerebrovascular inflammation. *Blood*.

[67] Loppnow H, Bil R, Hirt S (1998). Platelet-derived interleukin-1 induces cytokine production, but not proliferation of human vascular smooth muscle cells. *Blood*.

[68] Aslam R, Speck ER, Kim M (2006). Platelet Toll-like receptor expression modulates lipopolysaccharide-induced thrombocytopenia and tumor necrosis factor-*α* production in vivo. *Blood*.

[69] Clark SR, Ma AC, Tavener SA (2007). Platelet TLR4 activates neutrophil extracellular traps to ensnare bacteria in septic blood. *Nature Medicine*.

[70] Zhang G, Han J, Welch EJ (2009). Lipopolysaccharide stimulates platelet secretion and potentiates platelet aggregation via TLR4/MyD88 and the cGMP-dependent protein kinase pathway. *Journal of Immunology*.

[71] McMorran BJ, Marshall VM, De Graaf C (2009). Platelets kill intraerythrocytic malarial parasites and mediate survival to infection. *Science*.

[72] Alexander WS, Roberts AW, Nicola NA, Li R, Metcalf D (1996). Deficiencies in progenitor cells of multiple hematopoietic lineages and defective megakaryocytopoiesis in mice lacking the thrombopoietin receptor c-Mpl. *Blood*.

[73] Nørgaard M, Jensen AØ, Engebjerg MC (2011). Long-term clinical outcomes of patients with primary chronic immune thrombocytopenia: a Danish population-based cohort study. *Blood*.

[74] Kopp HG, Placke T, Salih HR (2009). Platelet-derived transforming growth factor-*β* down-regulates NKG2D thereby inhibiting natural killer cell antitumor reactivity. *Cancer Research*.

[75] Placke T, Kopp HG, Salih HR (2012). The wolf in sheep's clothing: platelet-derived, "pseudo self" impairs cancer cell, "missing self" recognition by NK cells. *Oncoimmunology*.

[76] Baenziger NL, Brodie GN, Majerus PW (1971). A thrombin-sensitive protein of human platelet membranes. *Proceedings of the National Academy of Sciences of the United States of America*.

[77] Crawford SE, Stellmach V, Murphy-Ullrich JE (1998). Thrombospondin-1 is a major activator of TGF-*β*1 in vivo. *Cell*.

[78] McMaken S, Exline MC, Mehta P (2011). Thrombospondin-1 contributes to mortality in murine sepsis through effects on innate immunity. *PLoS ONE*.

[79] Gawaz M, Dickfeld T, Bogner C, Fateh-Moghadam S, Neumann FJ (1997). Platelet function in septic multiple organ dysfunction syndrome. *Intensive Care Medicine*.

[80] Battinelli EM, Markens BA, Italiano JE (2011). Release of angiogenesis regulatory proteins from platelet alpha granules: modulation of physiologic and pathologic angiogenesis. *Blood*.

[81] Chatterjee M, Huang Z, Zhang W (2011). Distinct platelet packaging, release, and surface expression of proangiogenic and antiangiogenic factors on different platelet stimuli. *Blood*.

[82] Marrelli A, Cipriani P, Liakouli V (2011). Angiogenesis in rheumatoid arthritis: a disease specific process or a common response to chronic inflammation?. *Autoimmunity Reviews*.

[83] Grote K, Schütt H, Schieffer B (2011). Toll-like receptors in angiogenesis. *The Scientific World Journal*.

[84] Imhof BA, Aurrand-Lions M (2006). Angiogenesis and inflammation face off. *Nature Medicine*.

[85] Fiedler U, Reiss Y, Scharpfenecker M (2006). Angiopoietin-2 sensitizes endothelial cells to TNF-*α* and has a crucial role in the induction of inflammation. *Nature Medicine*.

[86] Scheuerer B, Ernst M, Dürrbaum-Landmann I (2000). The CXC-chemokine platelet factor 4 promotes monocyte survival and induces monocyte differentiation into macrophages. *Blood*.

[87] Austrup F, Vestweber D, Borges E (1997). P- and E-selectin mediate recruitment of T-helper-1 but not T-helper-2 cells into inflamed tissues. *Nature*.

[88] Diacovo TG, Puri KD, Warnock RA, Springer TA, Von Andrian UH (1996). Platelet-mediated lymphocyte delivery to high endothelial venules. *Science*.

[89] Elzey BD, Tian J, Jensen RJ (2003). Platelet-mediated modulation of adaptive immunity: a communication link between innate and adaptive immune compartments. *Immunity*.

[90] Elzey BD, Grant JF, Sinn HW, Nieswandt B, Waldschmidt TJ, Ratliff TL (2005). Cooperation between platelet-derived CD154 and CD4^+^ T cells for enhanced germinal center formation. *Journal of Leukocyte Biology*.

[91] Littman DR, Rudensky AY (2010). Th17 and regulatory T cells in mediating and restraining inflammation. *Cell*.

[92] Verschoor A, Neuenhahn M, Navarini AA (2011). A platelet-mediated system for shuttling blood-borne bacteria to CD8*α*
^+^ dendritic cells depends on glycoprotein GPIb and complement C3. *Nature Immunology*.

[93] Lang PA, Contaldo C, Georgiev P (2008). Aggravation of viral hepatitis by platelet-derived serotonin. *Nature Medicine*.

[94] Shalaby F, Rossant J, Yamaguchi TP (1995). Failure of blood-island formation and vasculogenesis in Flk-1 deficient mice. *Nature*.

[95] Stainier DYR, Weinstein BM, Detrich HW, Zon LI, Fishman MC (1995). cloche, an early acting zebrafish gene, is required by both the endothelial and hematopoietic lineages. *Development*.

[96] De Bruijn MFTR, Speck NA, Peeters MCE, Dzierzak E (2000). Definitive hematopoietic stem cells first develop within the major arterial regions of the mouse embryo. *EMBO Journal*.

[97] Chen AT, Zon LI (2009). Zebrafish blood stem cells. *Journal of Cellular Biochemistry*.

[98] Srinivasan RS, Dillard ME, Lagutin OV (2007). Lineage tracing demonstrates the venous origin of the mammalian lymphatic vasculature. *Genes and Development*.

[99] Bertozzi CC, Hess PR, Kahn ML (2010). Platelets: covert regulators of lymphatic development. *Arteriosclerosis, Thrombosis, and Vascular Biology*.

[100] Xu Y, Yuan L, Mak J (2010). Neuropilin-2 mediates VEGF-C-induced lymphatic sprouting together with VEGFR3. *Journal of Cell Biology*.

[101] Bertozzi CC, Schmaier AA, Mericko P (2010). Platelets regulate lymphatic vascular development through CLEC-2-SLP-76 signaling. *Blood*.

[102] Carramolino L, Fuentes J, García-Andrés C, Azcoitia V, Riethmacher D, Torres M (2010). Platelets play an essential role in separating the blood and lymphatic vasculatures during embryonic angiogenesis. *Circulation Research*.

[103] Suzuki-Inoue K, Inoue O, Ozaki Y (2011). Novel platelet activation receptor CLEC-2: from discovery to prospects. *Journal of Thrombosis and Haemostasis*.

[104] Echtler K, Stark K, Lorenz M (2010). Platelets contribute to postnatal occlusion of the ductus arteriosus. *Nature Medicine*.

[105] Rubinstein DB, Longo DL (1981). Peripheral destruction of platelets in chronic lymphocytic leukemia: recognition, prognosis and therapeutic implications. *American Journal of Medicine*.

[106] Berkman AW, Kickler T, Braine H (1984). Platelet-associated IgG in patients with lymphoma. *Blood*.

[107] Nieder C, Haukland E, Pawinski A, Dalhaug A (2010). Anaemia and thrombocytopenia in patients with prostate cancer and bone metastases. *BMC Cancer*.

[108] Ballot J, McDonnell D, Crown J (2003). Successful treatment of thrombocytopenia due to marrow metastases of breast cancer with weekly docetaxel. *Journal of the National Cancer Institute*.

[109] Chehal A, Taher A, Seoud M, Shamseddine A (2003). Idiopathic thrombocytopenic purpura and ovarian cancer. *European Journal of Gynaecological Oncology*.

[110] Rodeghiero F, Stasi R, Gernsheimer T (2009). Standardization of terminology, definitions and outcome criteria in immune thrombocytopenic purpura of adults and children: report from an international working group. *Blood*.

[111] Liebman H (2007). Other immune thrombocytopenias. *Seminars in Hematology*.

[112] Kaplan C, Ni H, Freedman J, Michelson AD (2011). Alloimmune thrombocytopenia. *Platelets*.

[113] Godeau B, Provan D, Bussel J (2007). Immune thrombocytopenic purpura in adults. *Current Opinion in Hematology*.

[114] Psaila B, Bussel JB (2008). Refractory immune thrombocytopenic purpura: current strategies for investigation and management. *British Journal of Haematology*.

[115] Abrahamson PE, Hall SA, Feudjo-Tepie M, Mitrani-Gold FS, Logie J (2009). The incidence of idiopathic thrombocytopenic purpura among adults: a population-based study and literature review. *European Journal of Haematology*.

[116] Cooper N, Bussel J (2006). The pathogenesis of immune thrombocytopaenic purpura. *British Journal of Haematology*.

[117] Cines DB, Bussel JB, Liebman HA, Luning Prak ET (2009). The ITP syndrome: pathogenic and clinical diversity. *Blood*.

[118] Stoll D, Cines DB, Aster RH, Murphy S (1985). Platelet kinetics in patients with idiopathic thrombocytopenic purpura and moderate thrombocytopenia. *Blood*.

[119] Ballem PJ, Segal GM, Stratton JR, Gernsheimer T, Adamson JW, Slichter SJ (1987). Mechanisms of thrombocytopenia in chronic autoimmune thrombocytopenic purpura. Evidence of both impaired platelet production and increased platelet clearance. *Journal of Clinical Investigation*.

[120] Gernsheimer T, Stratton J, Ballem PJ, Slichter SJ (1989). Mechanisms of response to treatment in autoimmune thrombocytopenic purpura. *New England Journal of Medicine*.

[121] Chang M, Nakagawa PA, Williams SA (2003). Immune thrombocytopenic purpura (ITP) plasma and purified ITP monoclonal autoantibodies inhibit megakaryocytopoiesis in vitro. *Blood*.

[122] Houwerzijl EJ, Blom NR, Van Der Want JJL (2004). Ultrastructural study shows morphologic features of apoptosis and para-apoptosis in megakaryocytes from patients with idiopathic thrombocytopenic purpura. *Blood*.

[123] Beardsley DS, Ertem M (1998). Platelet autoantibodies in immune thrombocytopenic purpura. *Transfusion and Apheresis Science*.

[124] Olsson A, Andersson PO, Tengborn L, Wadenvik H (2002). Serum from patients with chronic idiopathic thrombocytopenic purpura frequently affect the platelet function. *Thrombosis Research*.

[125] Yanagu M, Suzuki M, Soga T (1991). Influences of antiplatelet autoantibodies on platelet function in immune thrombocytopenic purpura. *European Journal of Haematology*.

[126] McMillan R, Wang L, Tomer A, Nichol J, Pistillo J (2004). Suppression of in vitro megakaryocyte production by antiplatelet autoantibodies from adult patients with chronic ITP. *Blood*.

[127] Yang L, Wang L, Zhao CH (2010). Contributions of TRAIL-mediated megakaryocyte apoptosis to impaired megakaryocyte and platelet production in immune thrombocytopenia. *Blood*.

[128] Olsson B, Andersson PO, Jernås M (2003). T-cell-mediated cytotoxicity toward platelets in chronic idiopathic thrombocytopenic purpura. *Nature Medicine*.

[129] Sayeh E, Sterling K, Speck E, Freedman J, Semple JW (2004). IgG antiplatelet immunity is dependent on an early innate natural killer cell-derived interferon-*γ* response that is regulated by CD8^+^ T cells. *Blood*.

[130] Oyaizu N, Yasumizu R, Miyama-Inaba M (1988). (NZW x BXSB)F1 mouse. A new animal model of idiopathic thrombocytopenic purpura. *Journal of Experimental Medicine*.

[131] Crow AR, Song S, Semple JW, Freedman J, Lazarus AH (2001). IVIg inhibits reticuloendothelial system function and ameliorates murine passive-immune thrombocytopenia independent of anti-idiotype reactivity. *British Journal of Haematology*.

[132] Nieswandt B, Bergmeier W, Rackebrandt K, Engelbert Gessner J, Zirngibl H (2000). Identification of critical antigen-specific mechanisms in the development of immune thrombocytopenic purpura in mice. *Blood*.

[133] Chow L, Aslam R, Speck ER (2010). A murine model of severe immune thrombocytopenia is induced by antibody- and CD8^+^ T cell-mediated responses that are differentially sensitive to therapy. *Blood*.

[134] Semple JW (2010). Animal models of immune thrombocytopenia (ITP). *Annals of Hematology*.

[135] Webster ML, Sayeh E, Crow M (2006). Relative efficacy of intravenous immunoglobulin G in ameliorating thrombocytopenia induced by antiplatelet GPIIbIIIa versus GPIb*α* antibodies. *Blood*.

[136] Cuker A, Cines DB (2010). Immune thrombocytopenia. *Hematology*.

[137] Go RS, Johnston KL, Bruden KC (2007). The association between platelet autoantibody specificity and response to intravenous immunoglobulin G in the treatment of patients with immune thrombocytopenia. *Haematologica*.

[138] Peng J, Ma S, Liu X (2011). Autoantibody to platelet GPIb/IX is a predictive factor for poor response to intrevenous immunoglobulin in adults with severe immune thrombocytopenia. *Journal of Thrombosis and Haemostasis*.

[139] Zeng Q, Zhu L, Tao L (2012). Relative efficacy of steroid therapy in immune thrombocytopenia mediated by anti-platelet GPIIbIIIa versus GPIbalpha antibodies. *American Journal of Hematology*.

[140] François B, Trimoreau F, Vignon P, Fixe P, Praloran V, Gastinne H (1997). Thrombocytopenia in the sepsis syndrome: role of hemophagocytosis and macrophage colony-stimulating factor. *American Journal of Medicine*.

[141] Peltier JY, Lambin P, Doinel C, Courouce AM, Rouger P, Lefrere JJ (1991). Frequency and prognostic importance of thrombocytopenia in symptom-free HIV-infected individuals: a 5-year prospective study. *AIDS*.

[142] Sloand EM, Klein HG, Banks SM, Vareldzis B, Merritt S, Pierce P (1992). Epidemiology of thrombocytopenia in HIV infection. *European Journal of Haematology*.

[143] Pawlotsky JM, Bouvier M, Fromont P (1995). Hepatitis C virus infection and autoimmune thrombocytopenic purpura. *Journal of Hepatology*.

[144] Pivetti S, Novarino A, Merico F (1996). High prevalence of autoimmune phenomena in hepatitis C virus antibody positive patients with lymphoproliferative and connective tissue disorders. *British Journal of Haematology*.

[145] Steeper TA, Horwitz CA, Moore SB (1989). Severe thrombocytopenia in Epstein-Barr virus-induced mononucleosis. *Western Journal of Medicine*.

[146] Wright JG (1992). Severe thrombocytopenia secondary to asymptomatic cytomegalovirus infection in an immunocompetent host. *Journal of Clinical Pathology*.

[147] Staub HP (1968). Postrubella thrombocytopenic purpura. A report of eight cases with discussion of hemorrhagic manifestations of rubella. *Clinical Pediatrics*.

[148] Yu XJ, Liang MF, Zhang SY (2011). Fever with thrombocytopenia associated with a novel bunyavirus in China. *New England Journal of Medicine*.

[149] Li Z, Nardi MA, Karpatkin S (2005). Role of molecular mimicry to HIV-1 peptides in HIV-1-related immunologic thrombocytopenia. *Blood*.

[150] Suerbaum S, Michetti P (2002). Helicobacter pylori infection. *New England Journal of Medicine*.

[151] Logan RPH, Walker MM (2001). ABC of the upper gastrointestinal tract: epidemiology and diagnosis of Helicobacter pylori infection. *British Medical Journal*.

[152] Gasbarrini A, Franceschi F, Tartaglione R, Landolfi R, Pola P, Gasbarrini G (1998). Regression of autoimmune thrombocytopenia after eradication of Helicobacter pylori. *The Lancet*.

[153] Inaba T, Mizuno M, Take S (2005). Eradication of Helicobacter pylori increases platelet count in patients with idiopathic thrombocytopenic purpura in Japan. *European Journal of Clinical Investigation*.

[154] Michel M, Cooper N, Jean C, Frissora C, Bussel JB (2004). Does Helicobater pylori initiate or perpetuate immune thrombocytopenic purpura?. *Blood*.

[155] Ando T, Tsuzuki T, Mizuno T (2004). Characteristics of Helicobacter pylori-induced gastritis and the effect of H. pylori eradication in patients with chronic idiopathic thrombocytopenic purpura. *Helicobacter*.

[156] Sullivan PS, Hanson DL, Chu SY, Jones JL, Ciesielski CA (1997). Surveillance for thrombocytopenia in persons infected with HIV: results from the multistate adult and adolescent spectrum of disease project. *Journal of Acquired Immune Deficiency Syndromes and Human Retrovirology*.

[157] Nardi MA, Liu LX, Karpatkin S (1997). GPIIIa-(49–66) is a major pathophysiologically relevant antigenic determinant for anti-platelet GPIIIA of HIV-1-related immunologic thrombocytopenia. *Proceedings of the National Academy of Sciences of the United States of America*.

[158] Nardi M, Tomlinson S, Greco MA, Karpatkin S (2001). Complement-independent, peroxide-induced antibody lysis of platelets in HIV-1-related immune thrombocytopenia. *Cell*.

[159] Zhang W, Nardi MA, Borkowsky W, Li Z, Karpatkin S (2009). Role of molecular mimicry of hepatitis C virus protein with platelet GPIIIa in hepatitis C-related immunologic thrombocytopenia. *Blood*.

[160] Yabu K, Kiyosawa K, Ako S (1994). Type C chronic hepatitis associated with thrombocytopenia in two patients. *Journal of Gastroenterology and Hepatology*.

[161] Adinolfi LE, Giordano MG, Andreana A (2001). Hepatic fibrosis plays a central role in the pathogenesis of thrombocytopenia in patients with chronic viral hepatitis. *British Journal of Haematology*.

[162] Herman JH, Jumbelic MI, Ancona RJ, Kickler TS (1986). In utero cerebral hemorrhage in alloimmune thrombocytopenia. *American Journal of Pediatric Hematology/Oncology*.

[163] Weiner E, Zosmer N, Bajoria R (1994). Direct fetal administration of immunoglobulins: another disappointing therapy in alloimmune thrombocytopenia. *Fetal Diagnosis and Therapy*.

[164] Gaddipati S, Berkowitz RL, Lembet AA, Lapinski R, McFarland JG, Bussel JB (2001). Initial fetal platelet counts predict the response to intravenous gammaglobulin therapy in fetuses that are affected by PLA1 incompatibility. *American Journal of Obstetrics and Gynecology*.

[165] Bussel JB, Zabusky MR, Berkowitz RL, McFarland JG (1997). Fetal alloimmune thrombocytopenia. *New England Journal of Medicine*.

[166] Ghetie V, Ward ES (2000). Multiple roles for the major histocompatibility complex class I-related receptor FcRn. *Annual Review of Immunology*.

[167] Roopenian DC, Akilesh S (2007). FcRn: the neonatal Fc receptor comes of age. *Nature Reviews Immunology*.

[168] McMillan R, Longmire RL, Tavassoli M, Armstrong S, Yelenosky R (1974). In vitro platelet phagocytosis by splenic leukocytes in idiopathic thrombocytopenic purpura. *New England Journal of Medicine*.

[169] Bussel JB, Primiani A (2008). Fetal and neonatal alloimmune thrombocytopenia: progress and ongoing debates. *Blood Reviews*.

[170] Kaplan C (2006). Foetal and neonatal alloimmune thrombocytopaenia. *Orphanet Journal of Rare Diseases*.

[171] Blanchette VS, Chen L, Salomon De Friedberg Z, Hogan VA, Trudel E, Decary F (1990). Alloimmunization to the Pl(A1) platelet antigen: results of a prospective study. *British Journal of Haematology*.

[172] Dreyfus M, Kaplan C, Verdy E, Schlegel N, Durand-Zaleski I, Tchernia G (1997). Frequency of immune thrombocytopenia in newborns: a prospective study. *Blood*.

[173] Bertrand G, Drame M, Martageix C, Kaplan C (2011). Prediction of the fetal status in noninvasive management of alloimmune thrombocytopenia. *Blood*.

[174] Mueller-Eckhardt C, Grubert A, Weisheit M (1989). 348 cases of suspected neonatal alloimmune thrombocytopenia. *The Lancet*.

[175] Murphy MF, Hambley H, Nicolaides K, Waters AH (1996). Severe fetomaternal alloimmune thrombocytopenia presenting with fetal hydrocephalus. *Prenatal Diagnosis*.

[176] Ghevaert C, Campbell K, Walton J (2007). Management and outcome of 200 cases of fetomaternal alloimmune thrombocytopenia. *Transfusion*.

[177] Kjeldsen-Kragh J, Killie MK, Tomter G (2007). A screening and intervention program aimed to reduce mortality and serious morbidity associated with severe neonatal alloimmune thrombocytopenia. *Blood*.

[178] Kaplan C, Daffos F, Forestier F (1988). Management of alloimmune thrombocytopenia: antenatal diagnosis and in utero transfusion of maternal platelets. *Blood*.

[179] Williamson LM, Hackett G, Rennie J (1998). The natural history of fetomaternal alloimmunization to the platelet- specific antigen HPA-1a (Pl^A1^, Zw^a^) as determined by antenatal screening. *Blood*.

[180] Kroll H, Kiefel V, Santoso S (1998). Clinical aspects and typing of platelet alloantigens. *Vox Sanguinis*.

[181] Bizzaro N, Dianese G (1988). Neonatal alloimmune amegakaryocytosis. Case report. *Vox Sanguinis*.

[182] Goldman M, Trudel E, Richard L, Khalife S, Spurll GM (2003). Neonatal alloimmune thrombocytopenia due to anti-HPA-2b (anti-Koa). *Immunohematology*.

[183] Kroll H, Kiefel V, Muntean W, Mueller-Eckhardt C (1994). Anti-KO^a^ as the cause of neonatal alloimmune thrombocytopenia. *Vox Sang*.

[184] Al-Sheikh IHA, Khalifa M, Rahi A, Qadri MI, Al Abad K (1998). A rare case of neonatal alloimmune thrombocytopenia due to anti-HPA-2b. *Annals of Saudi Medicine*.

[185] Grenet P, Dausset J, Dugas M, Petit D, Badoual J, Tangun Y (1965). Neonatal thrombopenic purpura with anti-Ko-a feto-maternal isoimmunization. *Archives Francaises de Pediatrie*.

[186] Davoren A, Curtis BR, Aster RH, McFarland JG (2004). Human platelet antigen-specific alloantibodies implicated in 1162 cases of neonatal alloimmune thrombocytopenia. *Transfusion*.

[187] Cines DB, Blanchette VS (2002). Medical progress: immune thrombocytopenic purpura. *New England Journal of Medicine*.

[188] McMillan R (2009). Antiplatelet antibodies in chronic immune thrombocytopenia and their role in platelet destruction and defective platelet production. *Hematology/Oncology Clinics of North America*.

[189] He R, Reid DM, Jones CE, Shulman NR (1994). Spectrum of Ig classes, specificities, and titers of serum antiglycoproteins in chronic idiopathic thrombocytopenic purpura. *Blood*.

[190] Kumpel B, King MJ, Sooranna S (2008). Phenotype and mRNA expression of syncytiotrophoblast microparticles isolated from human placenta. *Annals of the New York Academy of Sciences*.

[191] Kumpel BM, Sibley K, Jackson DJ, White G, Soothill PW (2008). Ultrastructural localization of glycoprotein IIIa (GPIIIa, *β*3 integrin) on placental syncytiotrophoblast microvilli: implications for platelet alloimmunization during pregnancy. *Transfusion*.

[192] Décary F, L’Abbé D, Tremblay L, Chartrand P (1991). The immune response to the HPA-1a antigen: association with HLA-DRw52a. *Transfusion Medicine*.

[193] Wu S, Maslanka K, Gorski J (1997). An integrin polymorphism that defines reactivity with alloantibodies generates an anchor for MHC class II peptide binding: a model for unidirectional alloimmune responses. *Journal of Immunology*.

[194] Parry CS, Gorski J, Stern LJ (2007). Crystallographic structure of the human leukocyte antigen DRA, DRB33^∗^0101: models of a directional alloimmune response and autoimmunity. *Journal of Molecular Biology*.

[195] Stuge TB, Skogen B, Ahlen MT, Husebekk A, Urbaniak SJ, Bessos H (2011). The cellular immunobiology associated with fetal and neonatal alloimmune thrombocytopenia. *Transfusion and Apheresis Science*.

[196] Ahlen MT, Husebekk A, Killie MK, Skogen B, Stuge TB (2009). T-cell responses associated with neonatal alloimmune thrombocytopenia: Isolation of HPA-1a-specific, HLA-DRB3^∗^0101-restricted CD4^+^ T cells. *Blood*.

[197] Ni H, Chen P, Spring CM (2006). A novel murine model of fetal and neonatal alloimmune thrombocytopenia: response to intravenous IgG therapy. *Blood*.

[198] Lang S, Yang H, Boyd S (2011). Impaired angiogenesis contributes to pathogenesis of fetal and neonatal immune thrombocytopenia. *Journal of Thrombosis and Haemostasis*.

[199] Schmaier AH (2011). Are maternal antiplatelet antibodies a prothrombotic condition leading to miscarriage?. *The Journal of Clinical Investigation*.

[200] Chen P, Li C, Lang S (2010). Animal model of fetal and neonatal immune thrombocytopenia: role of neonatal Fc receptor in the pathogenesis and therapy. *Blood*.

[201] Kaplan C (2010). FNAIT: the fetus pleads guilty!. *Blood*.

[202] Gonzalez CE, Pengetze YM (2005). Post-transfusion purpura. *Current Hematology Reports*.

